# Prevalence and characteristics of intended adolescent pregnancy: an analysis of the Canadian maternity experiences survey

**DOI:** 10.1186/s12978-015-0093-9

**Published:** 2015-11-05

**Authors:** Vineeth S. Sekharan, Theresa H. M. Kim, Elizaveta Oulman, Hala Tamim

**Affiliations:** School of Kinesiology and Health Science, York University, 4700 Keele Street, Toronto, ON Canada M3J 1P3

**Keywords:** Intended pregnancy, Adolescent pregnancy, Canada, Maternity experiences survey

## Abstract

**Background:**

There is limited research focusing on adolescent women who intended to become pregnant, as majority of research examines unintended adolescent pregnancies. The objective was to examine the prevalence and characteristics of Canadian adolescent women who intended to become pregnant.

**Methods:**

The analysis was based on the national 2006 Maternity Experiences Survey consisting of women who had a singleton live birth. The sample was restricted to adolescent women between 15 to 19 years of age. The main outcome of this study was the adolescent woman’s pregnancy intention. A variety of sociodemographic, maternal, and pregnancy related factors were examined using a multivariable logistic regression. Adjusted odds ratios (OR) and 95 % confidence intervals (CI) were reported for all variables.

**Results:**

The sample size was 290, weighted to represent 2224 adolescent women. Based on the adjusted model, the odds of experiencing an intended pregnancy were increased if the adolescent woman was between 18–19 years old (OR 2.62, 95 % CI 1.05, 6.57), had a partner (OR 2.37, 95 % CI 1.12, 4.99), experienced no violence/abuse (OR 3.08, 95 % CI 1.38, 6.86), and consumed no alcohol before pregnancy (OR 3.17, 95 % CI 1.56, 6.45). Additionally, adolescent women who reported drug use prior to pregnancy were more likely to have an intended pregnancy (OR 0.39, 95 % CI 0.16, 0.95).

**Conclusion:**

The findings from this study can be used as the basis for future research to investigate the characteristics and needs represented by this group of adolescents and to aid in the development of effective policies and programs.

## Background

Adolescent (or teenage) pregnancies have been documented to have adverse effects for both the mother and the child. Pregnant adolescents may face obstetric complications including preterm delivery, anemia, and hypertension [1]. Babies born to adolescent women may also experience health problems, such as premature birth and low-birth weight, severe neonatal conditions, and stillbirths [2–5]. Premature birth and low birth weight are also contributing factors to illnesses and conditions that are only recognized later in adulthood for the child, including epilepsy, mental retardation, autism, and dyslexia [1, 6, 7]. There tends to be high social and economic costs to teenage childbearing for both adolescents and society. Adolescent pregnancies often carry a high degree of social stigma depending on the social context, have higher sociodemographic risk factors, and pregnant adolescents are at increased risk of social isolation and abuse [8, 9]. The estimated difference in the cost with having a child before age 20 versus having a child at age 20 or 21 in the United States was 9.4 billion dollars [10].

According to the World Health Organization, 16 million adolescent girls give birth yearly, about 11 % of all worldwide births [11]. Teenage birth rate varies greatly between countries. For example, in the United States the teenage birth rate is 26.6 per 1000 women in 2013[12], while in Canada the teenage birth rate is 13.5 in 2010 [13]. Within countries, there exists variation in adolescent pregnancy rates across racial and ethnic groups, and regions. The highest teenage birth rates in Canada were reported by Nunavut (104.7 per 1000), the Northwest Territories (37.5), and Saskatchewan (34.4). The lowest teenage birth rates in Canada were reported in British Columbia (9.6), Quebec (9.8) and Ontario (10.5).

Almost all studies tend to focus only on unintended adolescent pregnancy and designing programs tailored to the needs of these women, and working towards taking steps for the prevention of future unintended adolescent pregnancy. However, despite the adverse effects strongly associated with adolescent pregnancy and childbirth, not all adolescent pregnancy is unwanted. A significant proportion of adolescent pregnancies is wanted and may even be planned. The proportion of intended pregnancies among adolescents varies greatly, and is lower in developed and wealthier countries like Canada and the United States [14]. In developing countries, proportion of planned pregnancies among pregnant adolescents ranges from 42 % in Columbia to 93 % in Egypt [14]. Studies conducted in the US noted the presence of racial differences between pregnant adolescents who did not want to become pregnant and pregnant adolescents who wanted to become pregnant, with increased representation by Hispanic-Americans and African-Americans (compared to Whites and Asians) in the latter group [15, 16]. Studies have also shown the distribution of unwanted and wanted adolescent pregnancy to correspond with differences in physical environment, population characteristics and cultural norms [16]. Adolescent intention of a pregnancy is important for both the wellbeing of the mother and the child, as unintended pregnancies are found to have adverse effects. A recent systematic review found that among unintended pregnancies, significantly increased odds of low birth weight and preterm births were reported [17]. Furthermore,lower pregnancy intention has been found to predict initial difficulties in parenting and high childbearing regret has been found to be reciprocally related across time with parenting stress [18].

To date, there has been no study conducted at the national level in Canada that looks at wanted pregnancies among adolescent girls. The objective of this study, therefore, is to examine the characteristics and traits of adolescent women throughout the provinces of Canada who wanted their pregnancy. Results can be used to create programs and develop supports tailored to the needs of adolescent women who wanted to become pregnant.

## Methods

### Database

Data was obtained from the secondary analysis of the 2006 Maternity Experiences Survey (MES), sponsored by the Public Health Agency of Canada and conducted by Statistics Canada. The MES utilizes a target population selected from the 2006 Canadian Census of Population. The birth cohort was selected from February 15, 2006 to May 15, 2006 for women living in the provinces and November 1, 2005 to February 1, 2006 for women living in the territories^1^. Women eligible to participate in the study included women who were at least 15 years of age at the time of the infant’s birth, who had a singleton live birth and lived with their infant during the time of their interview. Women excluded from the study were mothers who lived in First Nations reserves^2^ or institutions during the time of the survey. Data collection was obtained through a computer-assisted telephone interview, lasting approximately 45 min. Interviews were conducted by female professional Statistics Canada interviewers who were trained on the purpose of the study and protocol for questionnaire administration. Interviews were conducted 5 to 10 months post-partum for women living in the provinces and 9 to 14 months post-partum for women living in the territories. Ethics approval and informed consent was not required to obtain as this was not a clinical study and was based on secondary data analysis of the MES. The MES was presented to Health Canada’s Science Advisory Board, Health Canada’s Research Ethics Board, and the Federal Privacy Commissioner [19]. The design and methods of the MES has been previously described elsewhere [19]. For the purposes of this study, the present study sample was restricted to adolescent women, defined as women who were between 15 and 19 years of age at the time of the infant’s birth.

### Outcome

The main outcome of this study was the adolescent woman’s pregnancy intention. This variable was derived from the question “Thinking back to just before you became pregnant, would you say you wanted to be pregnant…?” and response categories included: sooner, then, later, or not at all. Women who reported wanting to become pregnant “sooner or then” were coded as having an intended/wanted pregnancy and women wanting to become pregnant “later or not at all” were coded as having an unintended/unwanted pregnancy.

### Independent variables

The potential characteristics for intended pregnancy among adolescent women included: 1) socio-demographic factors: age, aboriginal status, place of residence (urban versus rural), immigration to Canada, level of education, and presence of partner or significant other; 2) maternal health characteristics: previous depression diagnosis, and experience with violence over the last two years; and 3) pregnancy-related characteristics: cigarette smoking prior to pregnancy, alcohol use prior to pregnancy, and drug use prior to pregnancy. All of these variables, with the exception of experience with violence, were assessed using specific questions in the MES. The variable of experience with violence was based on a series of 10 questions about the mother’s experience with any form of physical or sexual violence. Women who responded yes to any question in the series were coded as having experienced violence or abuse.

### Statistical analysis

The prevalence of intended pregnancy among adolescent women was estimated through survey weights created by Statistics Canada and provided with the MES data set. Normalized weights, applied at the bivariate level were used to examine differences in the characteristics of intended pregnancy among adolescent women. Chi square tests were used to assess the association between the different levels of characteristics and intended pregnancy. Odds ratios (OR) and 95 % confidence intervals (95 % CI) were performed for all variables. To account for the complex sampling design, bootstrapping was performed to calculate all of the 95 % confidence estimates. A logistic multivariable analysis was performed for all variables found to be significant at the bivariate level. The sample sizes reported in this manuscript were derived from normalized weights, weighted to represent a larger population. All analyses were computed with Stata Data Analysis and Statistical Software (version 13.0), and set at alpha <0.05 for two-tailed test for statistical significance.

## Results

The sample size for the MES study analyzed was 290, weighted to represent 2224 Canadian adolescent women. Table 1 presents the unadjusted and adjusted associations between intended pregnancy and potential characteristics, among adolescent women. A variety of sociodemographic, maternal health, and pregnancy related variables were examined. Of the 11 variables examined in the analysis, only five variables remained significant in the adjusted model.

**Table 1:** Estimated prevalence and characteristics of an intended pregnancy among adolescent women based on a national survey of Canadian women (*N* = 290)

	Sample Size	Intended Pregnancy	Unadjusted Odds Ratio	Adjusted Odds Ratio
	N^a^	(%)	OR (95 % CI)^b^	OR (95 % CI)^b^
Socio-demographic Characteristics				
Age (years)**				
Old Teen (18–19 years)	214	32.6	3.20 (1.52, 6.73)	2.62 (1.05, 6.57)
Young Teen (15–17 years)	76	13.1	1	1
Aboriginal Status				
Aboriginal	48	24.9	0.88 (0.43, 1.79)	1.59 (0.60, 4.21)
Non-Aboriginal	238	27.4	1	1
Urban-rural residence*				
Urban, population ≤499,999	135	18.0	0.38 (0.19, 0.79)	0.45 (0.19, 1.05)
Urban, population ≥500,000	83	33.4	0.88 (0.41, 1.90)	1.48 (0.56, 3.92)
Rural area	52	36.3	1	1
Immigration to Canada				
Yes	18	41.6	2.03 (0.47, 8.73)	0.49 (0.05, 5.12)
No	268	26.0	1	1
Level of education				
No Education	46	26.1	0.95 (0.40, 2.27)	0.73 (0.25, 2.16)
High School	238	27.1	1	1
Partner/Significant other**				
Yes	146	35.0	2.38 (1.33, 4.27)	2.37 (1.12, 4.99)
No	139	18.5	1	1
Health-Related Characteristics				
Previous depression diagnosis				
No	238	29.2	1.89 (0.74, 4.79)	1.07 (0.38, 3.04)
Yes	50	17.9	1	1
Experienced violence within last 2 years**				
No	166	34.8	2.74 (1.47, 5.08)	3.08 (1.38, 6.86)
Yes	117	16.3	1	1
Pregnancy-Related Characteristics				
Cigarette smoking before pregnancy				
No	129	32.0	1.55 (0.88, 2.72)	1.05 (0.49, 2.22)
Yes	159	23.3	1	1
Alcohol use before pregnancy**				
No	114	39.0	2.66 (1.50, 4.69)	3.17 (1.56, 6.45)
Yes	173	19.4	1	1
Drug use before pregnancy				
No	216	26.1	0.80 (0.42, 1.53)	0.39 (0.16, 0.95)
Yes	72	30.6	1	1

*p-value < .05 between group analysis

**p-value < .01 between group analysis

^a^Sample size is estimated using normalized weights

^b^OR and 95 % CI were calculated using bootstrapping technique

Sociodemographic characteristics considered in the analysis of this study included the adolescent woman’s age, aboriginal status, urban–rural residence, immigration to Canada, level of education, and presence of a partner. Only two of these variables, age and presence of a partner, were found to be significant characteristics of intended pregnancy among adolescent women after adjusting for other variables. Results demonstrate that older adolescent women 18 to 19 years of age were more likely to report an intended pregnancy, compared to adolescent women who were 15 to 17 years of age (OR: 2.62; 95 % CI: 1.05, 6.57). Age remained a significant variable through adjustment; however, a weaker association was observed in the adjusted model. Additionally, adolescent women who reported having a partner or significant other were 2.37 times more likely to experience an intended pregnancy, compared to those who did not have a partner or significant other (95 % CI: 1.12, 4.99).

Analysis also considered a variety of maternal health characteristics, including previous depression diagnosis and experience with violence over the last two years. In the adjusted model, only experience with violence over the last two years was found to be a significant characteristic of experiencing an intended pregnancy among adolescent women. Women who did not report any experience with violence over the last two years were found to be 3.08 times more likely to have an intended pregnancy, compared to those who did experience violence or abuse (95 % CI: 1.38, 6.86).

Pregnancy related characteristics considered in the analysis included cigarette smoking, alcohol use and drug use prior to pregnancy. Analysis indicated that adolescent women who did not report drinking alcohol before pregnancy, were 3.17 times more likely to experience an intended pregnancy, compared to those who did use alcohol before pregnancy (95 % CI: 1.56, 6.45). Interestingly, analysis revealed that adolescent women who have used drugs prior to becoming pregnant were more likely to have an intended pregnancy, compared to those who did not use drugs prior to becoming pregnant (OR: 0.39; 95 % CI: 0.16, 0.95).

## Discussion

The present study was designed to investigate the prevalence and characteristics of adolescent women in Canada whose pregnancy was intended. The analysis mainly focused on pregnancy intention in relation to a host of maternal demographics, maternal health characteristics, and pregnancy-related statistics. According to a 2009 WHO document, 75 % of all adolescent pregnancies worldwide were intended [20]. The prevalence of intended adolescent pregnancy in the present study was 27.6 %, a significant portion of all adolescent women wanted to become pregnant. This is similar to figures obtained in a 1998 study on adolescent pregnancy conducted in California, in which 32 % of pregnant adolescent participants stated they had intended to become pregnant [15].

As compared to other adolescent women in the present study, adolescent women whose pregnancy was intended were more likely to fall into the older teen group (18–19 versus 15–17) and have a partner/significant other. They were less likely to have experienced violence within the last two years, or have used alcohol prior to their pregnancy. Surprisingly, adolescent women who had intended to become pregnant were more likely to have used drugs prior to their pregnancy.

Adolescent pregnancy increases the risk of adverse birth outcomes independent of known confounding factors, including maternal race, education, marital status, tobacco use, and alcohol use [1, 9]. This includes physical health risks for parent, including increased risk of maternal morbidity [21]. This also includes mental health risks, as pregnant and parenting adolescents are at greater risk of experiencing depressive symptoms than older pregnant and parenting adult women [22]. Results from the study showed that adolescent women who had intended their pregnancy were more likely to fall into the older teen group. Previous studies conducted in the United States have also shown that proportion of adolescent women who intended their pregnancies were older than those who had no intentions [15]. The increased desire for intended pregnancy among older teens compared to younger teens suggests recognition of reduced risks with increased age. The later teenage years, 18–19, are associated with a significant educational milestone for many teenagers in the completion of high school and may be a much more stable period for having a child.

Adolescent women who had not intended their pregnancy were significantly more likely to have experienced violence within the last two years and less likely to state that they had a partner when surveyed. While this study did not look closely at the relationship between intimate partner violence and pregnancy intention, the relationship between adolescent pregnancies and intimate partner violence has been widely documented in the literature [23–27]. An earlier study conducted in the US found that women with unwanted pregnancies were at increased risk for violence by their partners compared to women with intended pregnancies [28].

Partners play an important role in determining the intention, and the outcome, of a pregnancy [29–31]. In a recent study in the US, pregnant teenagers who had partner support were 63 % less likely to have a low birth weight child and two times less likely to lose their child compared to women who did not have partner support [30]. Perceived partner support predicts lower maternal and infant distress [32]. There are many potential reasons for this relationship. Partner support may mean improved financial outcomes for young mothers, as there are many costs associated with having a child, including costs associated with childcare, healthcare, and education. Partner support also provides expectant mothers with a means with which to mitigate the many social disadvantages commonly associated with adolescent pregnancy. Studies on pregnant women have shown that partner stability, status, dependability, and support have a moderating effect on women’s experiences of pregnancy [33, 34]. This moderating effect may operate at a greater level for adolescent girls compared to adult women. Perhaps most importantly, partner support may signify that parenting is a shared responsibility.

Adolescent women who had intended their pregnancies were less likely to have had consumed alcohol prior to their pregnancy than their peers. This may be because they are aware of health complications that can arise for both the child and the mother as a result of consuming alcohol (e.g., fetal alcohol syndrome). While adolescents with unintended pregnancies may also be aware of the harms of alcohol consumption for pregnant women, they are likely to be unaware that they were about to become pregnant. Indeed, preventing alcohol consumption prior to, and during pregnancies, is a primary focus of many campaigns targeting reproductive female health on the federal, provincial, and even the local level.

Adolescent women who had intended their pregnancies were significantly more likely to have used drugs prior to their pregnancy than adolescents who had not intended to become pregnant. This may be because adolescents who intended to become pregnant are less aware about the negative relationship that exists between drug consumption and pregnancy outcomes, compared to the awareness they may have about the relationship between alcohol consumption and poor pregnancy outcomes. One possible explanation is that drug use is the result of withdrawal symptoms experienced because of alcohol cessation. Future research examining drug use among adolescent women who intended to become pregnant may shed more light on this issue.

The results of this study should be cautiously interpreted as some limitations are imposed. One of the major limitations of the current study is that causality cannot be determined due to the cross-sectional nature of the study design. However, the cross-sectional nature of the study allowed us to determine the prevalence and explore characteristics of intended pregnancies among adolescent women. There is also a potential for recall bias however, interviews were generally conducted shortly after childbirth, which minimized the mother’s inability to respond accurately to questions referring back to their pregnancy. Despite such limitations, the study sample analyzed was representative of the Canadian population, with a high response rate of 75 %. Furthermore, to our knowledge, determining the prevalence and characteristics of intended pregnancies among adolescent women have not been conducted at the national level in Canada, and thus serves as an important stepping stone for future research considerations.

## Conclusions

The body of research available on intended adolescent pregnancy is very limited, despite a significant portion of adolescent pregnancies being intentional. Findings of this study serve as a reminder that adolescent mothers are not a homogeneous group, and that adolescent women who had intended to become pregnant may be living in a very different set of living circumstances, and accordingly have a different set of needs compared to adolescents whose pregnancies were not intended. More in depth qualitative and quantitative studies should aim to understand common traits possessed by this group of adolescent women, as well as their individual needs, to help inform and direct services and policies for adolescent mothers.

## Abbreviations

CI: Confidence Interval
MES: Maternity Experiences Survey
OR: Odds Ratio
US: United States
WHO: World Health Organization

## Footnotes

1 Canada has ten provinces (Alberta, British Columbia, Manitoba, New Brunswick, Newfoundland and Labrador, Nova Scotia, Ontario, Prince Edward Island, Quebec, and Saskatchewan), and three territories (Northwest Territories, Nunavut and Yukon). All of Canada’s territories are located in Northwestern Canada.

2 The term First Nations refers to Aboriginal peoples in Canada who are neither Inuit nor Metis.

## References

[1] Chen XK, Wen SW, Fleming N, Demissie K, Rhoads GG, Walker M (2007). Teenage pregnancy and adverse birth outcomes: A large population based retrospective cohort study. Int J Epidemiol.

[2] Fraser AM, Brockert JE, Ward RH (1995). Association of young maternal age with adverse reproductive outcomes. N Engl J Med.

[3] Gilbert W, Jandial D, Field N, Bigelow P, Danielsen B (2004). Birth outcomes in teenage pregnancies. J Matern Fetal Neonatal Med.

[4] Klein JD (2005). Adolescent pregnancy: Current trends and issues. Pediatrics.

[5] Harrison MS, Ali S, Pasha O, Saleem S, Althabe F, Berrueta M (2015). A prospective population-based study of maternal, fetal, and neonatal outcomes in the setting of prolonged labor, obstructed labor and failure to progress in low-and middle-income countries. Reprod health.

[6] Coley RL, Chase-Lansdale PL (1998). Adolescent pregnancy and parenting: Recent evidence and future directions. Am Psychol.

[7] Hellerstedt WL, Story M, Stang J (2002). Economic, psychosocial, and health risks associated with adolescent childbearing. Nutrition and the pregnant adolescent: A practical reference guide.

[8] Wiemann CM, Vaughn IR, Berenson AB, Volk RJ (2004). Are pregnant adolescents stigmatized by pregnancy?. J Adolesc Health.

[9] Fleming N, Ng N, Osborne C, Biederman S, Yasseen AS, Dy J (2013). Adolescent pregnancy outcomes in the province of Ontario: a cohort study. J obstet gynaecol Can: JOGC = Journal d'obstetrique et gynecologie du Canada: JOGC.

[10] The National Campaign to Prevent Teen and Unplanned Pregnancy. Counting it up: The public costs of teen childbearing: Key data. Washington, DC. http://thenationalcampaign.org/press-release/teen-childbearing-cost-taxpayers-94-billion-2010. Accessed 11 August 2015.

[11] World Health Organization (WHO) (2012). Born too soon: The global action report on preterm birth.

[12] Hamilton BE, Martin JA, Osterman MJK, Curtin SC. Births: Preliminary Data for 2013. Hyattsville, MD: National Center for Health Statistics. 2014. http://www.cdc.gov/nchs/data/nvsr/nvsr63/nvsr63_02.pdf. Accessed 11 August 2015.

[13] McKay A (2013). Trends in Canadian National and Provincial/Territorial Teen Pregnancy Rates: 2001–2010. Can J Hum Sex.

[14] World Health Organization (WHO) (2010). Position paper on mainstreaming adolescent pregnancy in efforts to make it safer.

[15] Frost JJ, Oslak S (1999). Teenagers’ pregnancy intentions and decisions: a study of young women in California choosing to give birth. Occasional Report. No. 2.

[16] Unger JB, Molina GB, Teran L (2003). Perceived consequences of teenage childbearing among adolescent girls in an urban sample. Soc Sci Med.

[17] Shah PS, Balkhair T, Ohlsson A, Beyene J, Scott F, Frick C (2011). Intention to become pregnant and low birth weight and preterm birth: a systematic review. Matern Child Health J.

[18] East PL, Chien NC, Barber JS (2012). Adolescents’ Pregnancy intentions, wantedness, and regret: cross-lagged relations with mental health and harsh parenting. J Marriage Fam.

[19] Dzakpasu S, Kaczorowski J, Chalmers B, Heaman M, Duggan J, Neusy E (2008). Maternity Experiences Study Group of the Canadian Perinatal Surveillance System, Public Health Agency of Canada. The Canadian maternity experiences survey: Design and methods. J Obstet Gynaecol Can: JOGC = Journal d'Obstetrique Et Gynecologie Du Canada : JOGC.

[20] Department of Making Pregnancy Safer, World Health Organization (2008). Adolescent pregnancy. Making Pregnancy Safe Notes.

[21] Dangal G. An update on teenage pregnancy. Int J Gynecol Obstet. 2004;5(1).

[22] Siegel RS, Brandon AR (2014). Adolescents, pregnancy, and mental health. J Pediatr Adolesc Gynecol.

[23] O’Donnell L, Agronick G, Duran R, Myint UA, Stueve A (2009). Intimate partner violence among economically disadvantaged young adult women: associations with adolescent risk-taking and pregnancy experiences. Perspect Sex Reprod Health.

[24] Pallitto CC, Campbell JC, O’Campo P (2005). Is intimate partner violence associated with unintended pregnancy?: A review of the literature, Trauma. Violence & Abuse.

[25] Miller E, Decker MR, McCauley HL, Tancredi DJ, Levenson RR, Waldman J (2010). Pregnancy coercion, intimate partner violence and unintended pregnancy. Contraception.

[26] Silverman JG, Raj A, Mucci L, Hathaway J (2001). Dating violence against adolescent girls and associated substance use, unhealthy weight control, sexual risk behavior, pregnancy, and suicidality. J Am Med Assoc.

[27] Roberts TA, Auinger P, Klein JD (2005). Intimate partner violence and the reproductive health of sexually active girls. J Adolesc Health.

[28] Gazmararian JA, Adams MM, Saltzman LE, Johnson CH, Bruce FC, Marks JS (1995). The relationship between pregnancy intendedness and physical violence in mothers of newborns. The PRAMS Working Group. Obstet Gynecol.

[29] Ney P, Peeters-ney MA, Fung T, Sheils C (2013). How partner support of an adolescent affects her pregnancy outcome. Webmed Central Public Health.

[30] Shah MK, Gee RE, Theall KP (2014). Partner support and impact on birth outcomes among teen pregnancies in the United States. J Pediatr Adolesc Gynecol.

[31] Alio AP, Mbah AK, Grunsten RA, Salihu HM (2011). Teenage pregnancy and the influence of paternal involvement on fetal outcomes. J Pediatr Adolesc Gynecol.

[32] Stapleton LR, Schetter CD, Westling E, Rini C, Glynn LM, Hobel CJ (2012). Perceived partner support in pregnancy predicts lower maternal and infant distress. J Fam Psychol.

[33] Kroelinger CD, Oths KS (2000). Partner support and pregnancy wantedness. Birth.

[34] Unger DG, Wandersman LP (1988). The relation of family and partner support to the adjustment of adolescent mothers. Child Dev.

